# 

**Published:** 1870-04

**Authors:** 


CONTENTS:

ORIGINAL COMMUNICATIONS:

ART. I. Upon Certain Mechanical Means of Restoring a Retroverted Uterus. By H. Webster Jones, A.M., M.D. - 193
" II. Medical Domain of the Turkish Bath. By M. P. Hanson, M.D. - - - - - 201
" III. Musk in Hiccough. By J. E. O'Brien, M.D., - - - - 205
" IV. Amputation of the Right Arm at the Shoulder Joint, with incision of a portion of the Shoulder Blade for a Necrosis. By W. H. Hess, M.D. 207
" V. Abnormal Situation of the Anus with Intestinal Calculi. By B. C. Brett, M.D. - - 208
" VI. Addendum to Article of H. Culbertson, M.D., - - - 210
" VII. Report of a case of Diphtheria, followed by Epilepsy. By N. Bridge, M.D., - - 211

CORRESPONDENCE:

"Form and Motion." By Z. C. McElroy, M.D., - - - - - - - - - - - - 215

FOREIGN ITEMS,

I. Case of Spontaneous Popliteal Aneurism. - - - - - - - - 218
II. Influence of Electricity upon Parturition. - - - - - - - - - 218
III. Injection of the constituents of bile into the veins of an animal. 219
IV. Tarter-Emetic in Pneumonia. - 221
V. Operation for Cataract. - - - 222
VI. Case of Epilepsy. - - - - - 222

SELECTED ARTICLES:

ART. 1. On Relapsing Fever. A lecture by Austin Flint, M.D. 223

EDITOR'S BOOK TABLE:

Flint's Physiology of Man. - - - - 240
Tyson's Cell Doctrine. - - - - - - 241
Naphey's Modern Therapeutics. - - 242
Hall's Health by Good Living. - - - 242

EDITORIAL,

Sale of Diplomas. - - - - - - - 248
Cook Co Hospital. - - - - - - - 248

MEDICAL GOSSIP, - - - - - - - - - 248

Cerebro-Spinal Meningitis, - - - - 256
American Medical Association, - - - 256

